# An Uncommon Cause of Dysuria in a Female Patient: Huge Urinary Bladder Stones

**DOI:** 10.7759/cureus.1788

**Published:** 2017-10-20

**Authors:** Rizwan Ishtiaq, Afzal Randhawa, Laraib Zulfiqar, Naila Shabbir

**Affiliations:** 1 Gastroenterology, Beth Israel Deaconess Medical Center, Boston; 2 General Surgery, Bahawal Victoria Hospital, Bahawalpur, Pakistan; 3 General Medicine, Quaid-e-Azam Medical College, Bahawalpur, Pakistan; 4 Obstetrics & Gynecology, Bahawal Victoria Hospital, Bahawalpur, Pakistan

**Keywords:** dysuria, urinary bladder, stones

## Abstract

The urinary bladder stones are formed due to urinary retention, obstruction to the flow of urine commonly caused by enlargement of the prostate in males, urinary tract infections, and foreign body. The urinary bladder stones are usually found in males, and its presentation in females is a rare entity. Recurrence of urinary tract stones is commonly due to either repeated urinary tract infections or any metabolic condition. We present a case of a 75-year-old female patient who had a history of urinary tract infection and bladder stone formation and was operated for the 16th time recently for removal of her bladder stones.

## Introduction

The urinary bladder stones account for 5% of all the urinary tract lithiasis, and giant urinary bladder calculi are defined as stones with weight more than 100 g and dimensions larger than 4 cm in diameter [1]. The bladder stones are more common in males, and usually, it is an alarming sign of urinary retention, urinary obstruction, foreign body retention, urinary tract infection or enlargement of the prostate in males [2]. Diet and hydration status of the body also play an important role [2]. Open cystostomy, shock wave lithotripsy, and transurethral lithotripsy are well-known procedures for removal of the urinary bladder stones. Urinary bladder stones are the rare phenomenon in females. We hereby present a case of urinary bladder stones in a female patient with a past medical history of the urinary tract infections and bladder stones. Informed consent statement was obtained from the patient for this study.

## Case presentation

A 75-year-old female presented to the emergency department with complaints of abdominal pain, burning micturition, dysuria and hematuria for the last two weeks. The patient described it as a feeling of heaviness in the perianal region. On questioning, the patient reported that such complaints had been occurring for the last 20 years and she has been operated 15 times for stones in the urinary bladder by her primary care physicians in the village. Also, she had received multiple treatments for her episodes of urinary tract infections during this time. The patient's diet composed of food with high levels of oxalate, spinach, and red meat. She also reported drinking poor water as the village had no access to clean water source.

**Figure 1:**
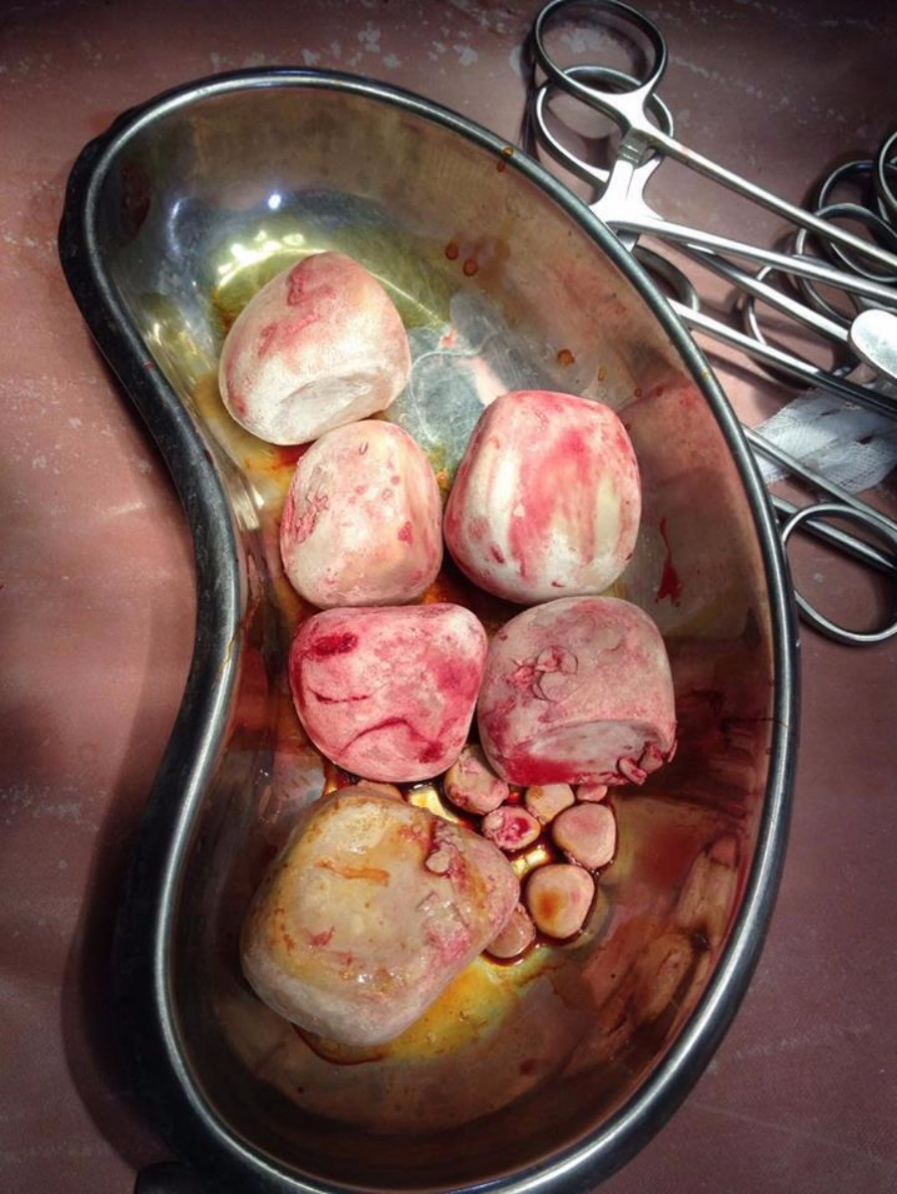
The urinary bladder stones removed during the surgery of the patient.

During the physical examination, a non-tender lump was palpable in the suprapubic region. The urinalysis test was consistent with hematuria and pyuria. Remaining investigations, including the renal function tests, were within the normal limits. The X radiation (X-ray) showed six large calcifications, each measuring 5.5x4.5 cm along with multiple small calcifications in the pelvic cavity. Ultrasonography revealed bilateral hydronephrosis along with multiple giant vascular calculi. Cystoscopy performed before the surgery showed round and oval urinary stones. Cystolithotomy was performed and stones were removed. The combined weight of all the stones was approximately 450 g. The stones were not adherent to the bladder mucosa. The primary bladder incision was closed with two layers of 2.0 catgut sutures. No urethral obstruction was found. Through the urethra, Foley’s catheter was inserted. Figure 1 reveals the stones removed from the urinary bladder of the patient.

The patient’s postoperative course was unremarkable and she was discharged on the 10th postoperative day. The Foley's catheter was removed on the 10th postoperative day, and bilateral hydronephrosis was found to be resolved on ultrasound. The patient had no complaints in voiding despite complaining of irritative lower urinary tract symptoms. The patient lost to follow-up, so the stone analysis and workup for any metabolic disorder could not be performed.

## Discussion

The urinary stones in female patients are a rare phenomenon. Ninety-five percent of the urinary stones are found in the male patients [3]. Our patient came from a village where people had no access to safe drinking water and lacked necessary diagnostic facilities in the area. The patient's medical history accounts of multiple urinary tract infections and lack of access to safe drinking water source may have resulted in large stones in the patient. In addition to this, any undiagnosed metabolic syndrome can be a cause of recurrent stone in the patient. Due to loss to follow-up by the patient, we were unable to diagnose any metabolic condition which may have caused the repeated formation of stones.

The formation of stones is a prolonged process resulting from the supersaturation of the solubility product in the urinary stream. Once the supersaturation exceeds the critical limit, the crystal precipitates out [4]. Typical components of urine like calcium, oxalate, uric acid, and phosphate make up most of the calculi formed in the urinary system. Repeated urinary tract infections caused by urea-splitting bacteria commonly Proteus mirabilis which leads to an alteration in the pH of the urine is a common cause of struvite stones [5]. A history of suprapubic pain dysuria, weak urinary stream and hematuria may be helpful in the diagnosis of stones in the urinary tract but are not pathognomonic since tumors, and other lesions in the bladder may cause similar signs and symptoms.

The X-ray and ultrasound are helpful in the diagnosis of stones in the urinary bladder. Ninety percent of the stones are made up of calcium phosphate and calcium oxalate, so they are radiopaque on the plain abdominal radiograph [6]. Less radiopaque stones include cysteine, uric acid or struvite stones [6]. The choice of treatment depends on the cause and size of the calculus. For stones smaller than 2 cm in size, the extracorporeal lithotripsy appears to show a good result [7]. For stones larger than 2 cm in size, the surgery is the primary treatment option. Cystolithotomy is the preferred method of removing sizeable urinary bladder stones from the body [2].

The large size of the bladder stone can cause urinary stasis leading to hydronephrosis and eventually renal failure. We found two cases of renal failure caused by giant bladder stones in the female patients [8]. Although our patient did not suffer any complication from the giant urinary balder stones, we recommend immediate treatment in the short term to avoid further complications.

## Conclusions

We present the case of a female patient with multiple episodes of urinary tract infection and urinary bladder stones in the past. It was her 16th operation for the removal of urinary bladder stones. We recommend that physicians should be able to rule out the possibility of the urinary stones in any patient with a significant history of urinary infections and urinary calculi, presenting with urinary complaints. The treatment should not be delayed to prevent any possible complications. Renal function tests should be regularly followed for such patients. In cases of recurrent stones, regular medical workup of the body should be done to rule out any enzyme deficiency or any undiagnosed metabolic syndrome.
